# Resonant Ring with a Gain of 36 for Use with a 1 MW 110 GHz Gyrotron

**DOI:** 10.1007/s10762-024-00991-0

**Published:** 2024-05-31

**Authors:** Elliot L. Claveau, Michael A. Shapiro, Richard J. Temkin

**Affiliations:** 1 Plasma Science and Fusion Center, Massachusetts Institute of Technology, 167 Albany St, Cambridge, MA 02139, USA

**Keywords:** Ring resonator, Gyrotron, Laser driven semiconductor switch, Pulse compressor

## Abstract

A 110 GHz quasi-optical ring resonator, designed for use with a 1 MW pulsed gyrotron, has been built and successfully tested using a 100 mW solid-state source. A low reflectance (2.4%) input coupler and a low-loss, four-mirror ring demonstrated a compression ratio, defined as the ratio of output to input power, of 36. The 6 ns output pulses were generated from the 2 m length ring using a silicon laser-driven semiconductor switch (LDSS). The quasi-optical ring resonator was designed with large waist sizes so that input pulses of up to 1 MW will stay under the 35 kV/cm electric field limit for ionization in ambient air. Maximum compression gain was achieved by matching the input coupling fraction to the round trip loss in the ring, achieving close to critical coupling. The experimental output pulse shape obtained after firing the LDSS was modeled using the reflectance, transmittance, and absorptance of the switch vs. time and vs. laser pulse fluence, with good agreement found with theory. The timing for the peak energy efficiency of 32% was found and the main loss mechanism limiting that efficiency was found to be the absorptance in the silicon wafer.

## 1 Introduction

High-power sources in the MMW/THz range with pulse lengths on the nanosecond scale are challenging to engineer. At low power, solid-state sources can provide 10 to 100 mW, but higher power levels are harder to achieve. The range of tens of watts is necessary to obtain an adequate signal-to-noise ratio in dynamic nuclear polarization nuclear magnetic resonance (DNP-NMR) techniques [1, 2]. At the high-power level, gyrotron sources can provide MW-level output power both in cw and pulsed operation, but ring resonators [3, 4] have the potential to simultaneously shorten the pulses and increase the power for suitable applications such as powering high gradient linear particle accelerators [5, 6].

Previous compression/gain techniques have been investigated for frequencies of up to 35 GHz using delay lines or microwave cavities directly attached to waveguides [7–11]. Quasi-optical resonant rings have been investigated without any ways to extract the trapped power [12–14]. These aimed to test components inserted in the path of the ring. Fully operational ring resonators with an active output coupler have been previously built using plasma switches or phase manipulation [7, 15, 16]. These previous experiments showed limited gain. Recently, a 250 GHz ring resonator with a gain of 16 [17], a 95 GHz ring resonator with a gain of 37 [18], and a 170 GHz ring resonator with a gain of about 25 [19] have been demonstrated. These concepts used a quasi-optical ring with a Laser Driven Semiconductor Switch (LDSS) employed as the output coupler. LDSSs have been experimentally demonstrated [20–26] and a robust theory describes their most important characteristics [27, 28].

The present work demonstrates the use of a ring resonator compatible with input powers of up to 1 MW with a gain of 36. This is possible by reducing coupling losses through a single fused silica input coupler. Also, length-tuning capabilities are implemented in the ring resonator design to compensate for temperature effects.

The beam waist is kept large in order to stay below the 35 kV/cm electric field ionization limit in the atmosphere [29]. Moreover, MW-level operation was demonstrated in [23], which showed a small increase in LDSS reflectance when operating at high 110 GHz power levels. Therefore, the pulse compressor could operate at a modestly higher gain value due to the increase in reflectance when using a 1 MW beam.

We investigate the output pulse theoretically by developing an algorithm using the LDSS physics to successfully reproduce the output pulse shape. This output pulse shape is further investigated experimentally by varying the laser energy density. We also show how measurements of the input and output signals can be used to predict important characteristics such as the gain, the losses, and the Q values. Finally, the energy efficiency of the system is investigated and the main source of losses is found.

**Fig. 1:**
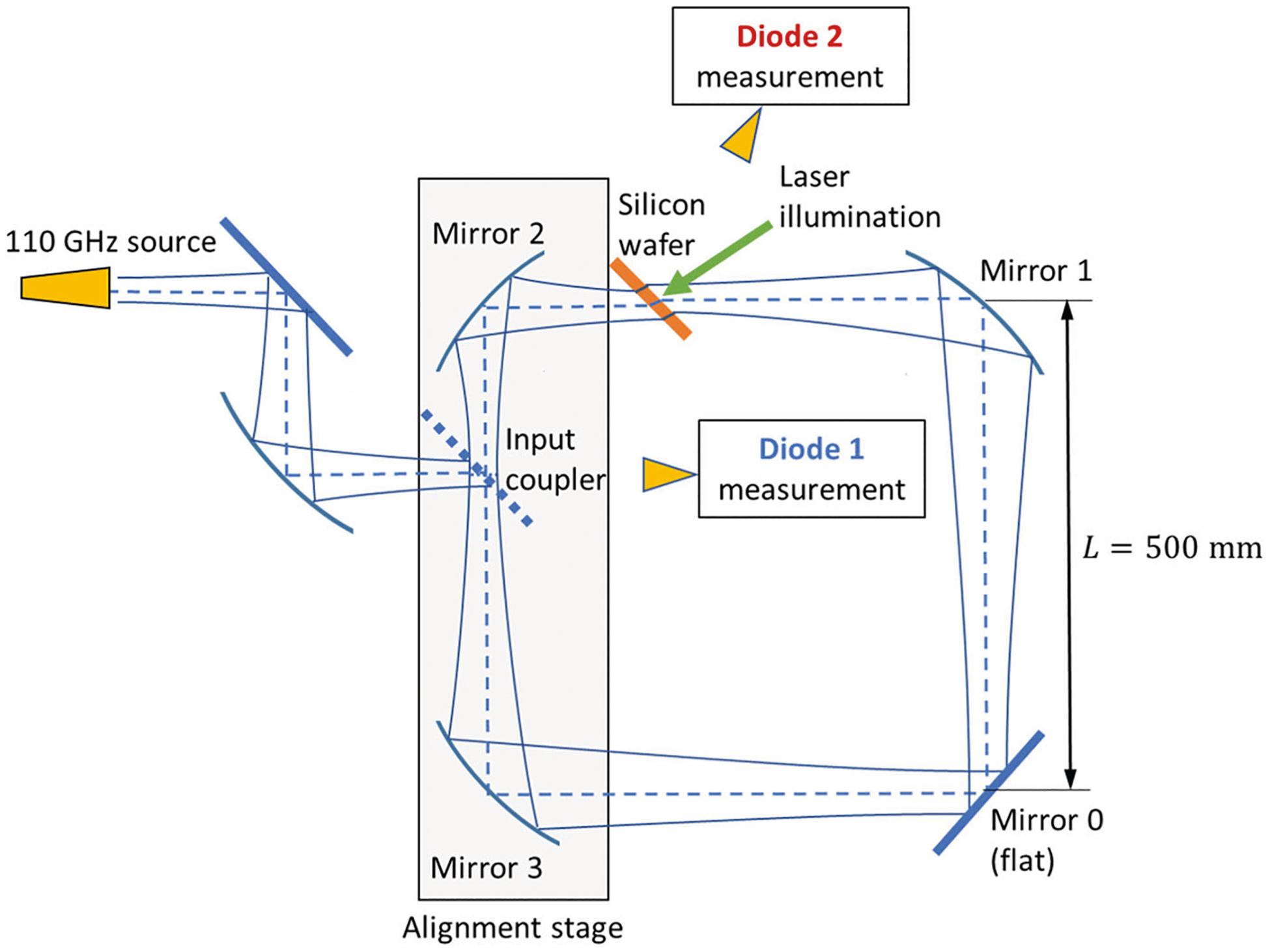
Schematic of the ring resonator using three shaped mirrors and one flat mirror. Diode 1 measures a combination of the input signal passing through the coupler with a fraction of the trapped circulating power reflected out by the coupler. Diode 2 measures the ring resonator output

Figure 1 shows the experimental setup consisting of 3 ellipsoidal mirrors, one flat mirror, an input coupler, and an output coupler. The output coupler is a laser-driven semiconductor switch. Mirrors 0 through 3 form a 2 m long ring. The input coupler is a fused silica, 803μm thick wafer that reflects 2.4% of the incident power at 110 GHz into the ring. The input beam is generated at a solid-state source and then passed through a corrugated horn generating a Gaussian beam.

This beam is then expanded and focused by an ellipsoidal mirror onto the input coupler to form a 32 mm waist. This large waist guarantees that the electric field stays below the ionization limit near the mirror surfaces, where the field is doubled. This design choice should allow the operation in-air with a 1 MW source, avoiding the use of a complex vacuum system. Moreover, the resulting large confocal distance and corresponding large focal lengths of the ring mirrors reduce the cross-polarization losses [30].

**Fig. 2:**
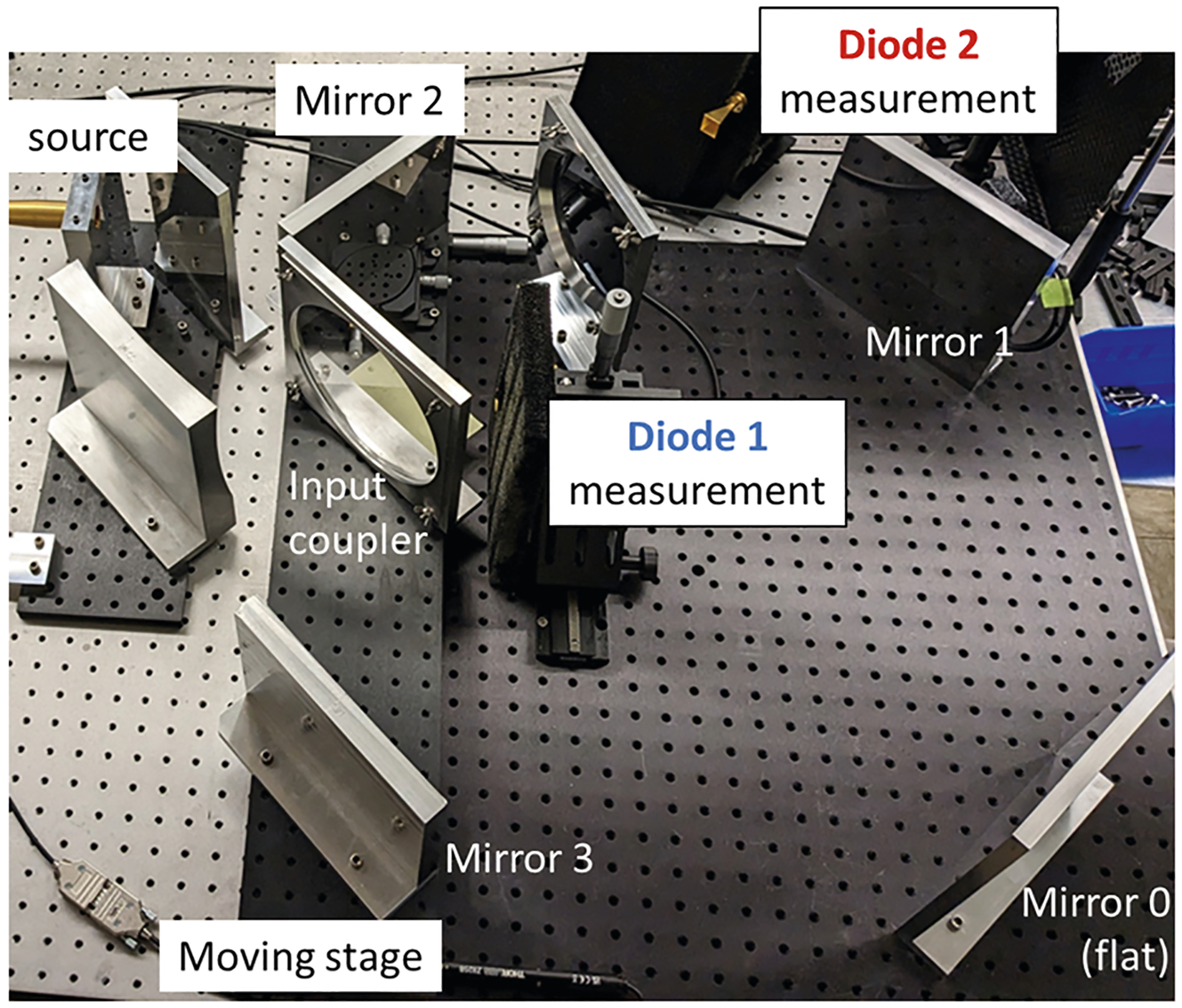
Photo of the experimental setup. The ring is tuned by moving the platform on which Mirror 2, Mirror 3, and the input coupler are located. This moving stage can change the ring length without affecting the alignment. The source is at the left and is not visible in the photo

In the present 110 GHz ring resonator, Mirrors 2 and 3 are placed on an alignment stage as shown on Fig. 2. This stage can finely adjust the ring length, thus compensating for temperature changes without having to adjust the frequency.

Care was also taken to achieve close to critical coupling in the 110 GHz ring. The input coupler was chosen to have a reflectivity equal to the total estimated ring round trip losses. This maximizes the gain of the ring.

## 2 Results

Measurements from Diode 1 show the charging physics and are used to calculate Q values as well as a range of internal peak gain. Those calculated quantities are further refined by using Diode 2 measurements along with an algorithm developed to accurately predict the output pulse shape. Then, we investigate how the output pulse shape evolves with laser energy, before finally investigating the energy efficiency of the experiment.

### 2.1 Ring Charging

**Fig. 3:**
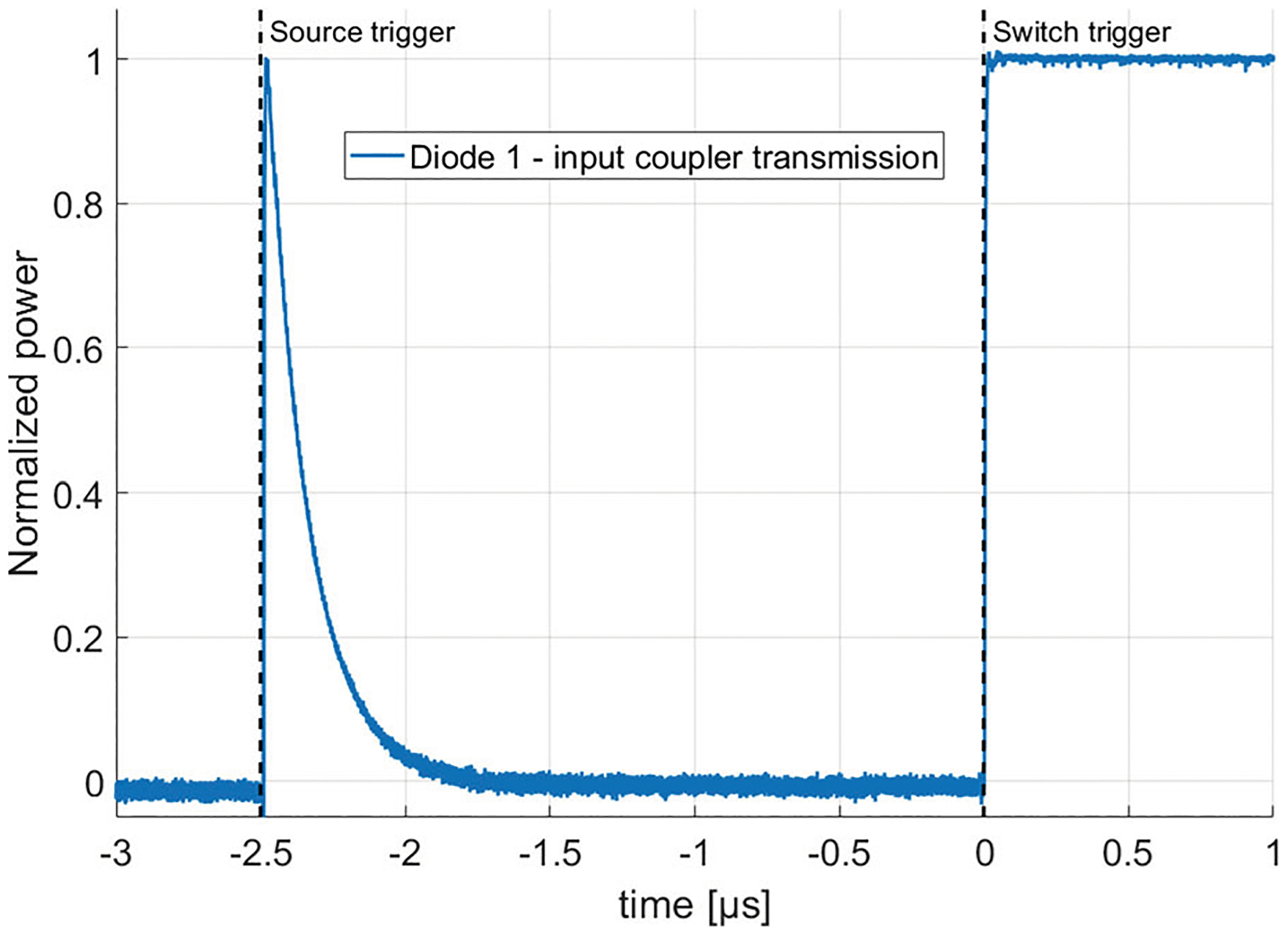
Filling of the ring as measured by Diode 1. An exponential function is fitted to the time trace right after the source triggers in order to infer the Q value of the ring. The reference value for the incident power is obtained after the switch triggers

Figure 3 shows the time evolution of the ring power as measured by Diode 1. Diode 1 measures the combination of the incoming source power and the reflected ring power. When tuned, those two signals are out of phase such that they destructively interfere. The tuning process involves either adjusting the source frequency or modifying the ring resonator’s total length via the moving stage such that an integer number of wavelengths are present inside the ring.

At this resonance condition, the charging of the ring looks like a decreasing exponential function saturating at a low value. This charging period is described by the following equation [4]:

(1) $$\frac{P_{\text{ring}}(t)}{P_{0}}=\frac{\omega_{0}c}{Q_{c}L}{\left(\frac{2Q_{tot}}{\omega_{0}}\right)}^{2}{\left(1-\text{exp}\left(-\frac{\omega_{0}t}{2Q_{tot}}\right)\right)}^{2}$$

where $P_{\text{ring}}$ is the power stored inside the ring, $P_{0}$ is the source power. $Q_{tot},\;Q_{c}$, and $Q_{\text{loss}}$ are the total, coupling, and loss Q values, which are defined as

(2) $$Q_{c}=\frac{\omega_{0}L}{R_{in}^{2}c}$$

(3) $$Q_{\text{loss}}=\frac{\omega_{0}L}{\alpha c}$$

(4) $$Q_{\text{tot}}=\frac{Q_{\text{loss}}Q_{c}}{Q_{\text{loss}}+Q_{c}}$$

where $\omega_{0}/2\pi$ is the input frequency of 110 GHz, L is the ring length of 2 m, c is the speed of light, $R_{\text{in}}$ is the input coupler reflection coefficient, and α is the ring power loss per round trip.

After the power in the ring reaches equilibrium, the value of the power $P_{tr}$ measured at Diode 1 is given by Eq. 5 [4]:

(5) $$\frac{P_{tr}}{P_{0}}={\left(1-\frac{2Q_{tot}}{Q_{c}}\right)}^{2}.$$

A value of $Q_{tot}=(9.7\pm 0.3)\times 10^{4}$ is obtained by fitting the portion of the curve right after the source trigger in Fig. 3 using Eq. 1. Then, using Eq. 5, the ratio of $Q_{tot}/Q_{c}$ is calculated from the ratio of the average Diode 1 value before the switch trigger $\left(P_{tr}\right)$ to the transmitted power after the switch trigger $\left(P_{0}\right)$. This calculated ratio has larger error bars because of the small measured value of $P_{tr}$. Due to the inherent calibration uncertainty as well as the electronic noise, the estimated value of $Q_{c}$ is (2.2 ± 0.3) × 10^5^ yielding a calculated ratio of $Q_{tot}/Q_{c}=0.45\pm 0.05$. The measured value of $Q_{tot}/Q_{c}$ is close to the value of 0.50, which corresponds to the case of “critical coupling”. Critical coupling is the case where $Q_{c}=Q_{\text{loss}}$ and is the optimal design point for maximizing the gain of a ring resonator. In that case, the electric field transmitted through the input coupler from the source and the field reflected out of the ring by the input coupler completely destructively interfere. Therefore, Diode 1 measures no power. This is the intended design of the present resonator.

Knowing the ratios and the value of $Q_{tot}$, a gain is calculated using Eqs. 2 – 4 as well as the following equation derived from Eq. 1:

(6) $$Gain(t\to \infty )=\frac{1}{\alpha }\frac{4Q_{\text{loss}}/Q_{c}}{{\left(Q_{\text{loss}}/Q_{c}+1\right)}^{2}}$$

Equation 6 calculates a gain from Diode 1 of 38 ± 7. This range of values corresponds to the maximum attainable gain inside the ring. If the switch were perfectly reflective, this would correspond to the gain measured by Diode 2 after triggering the LDSS.

### 2.2 Theoretical Output Pulse Shape

**Fig. 4:**
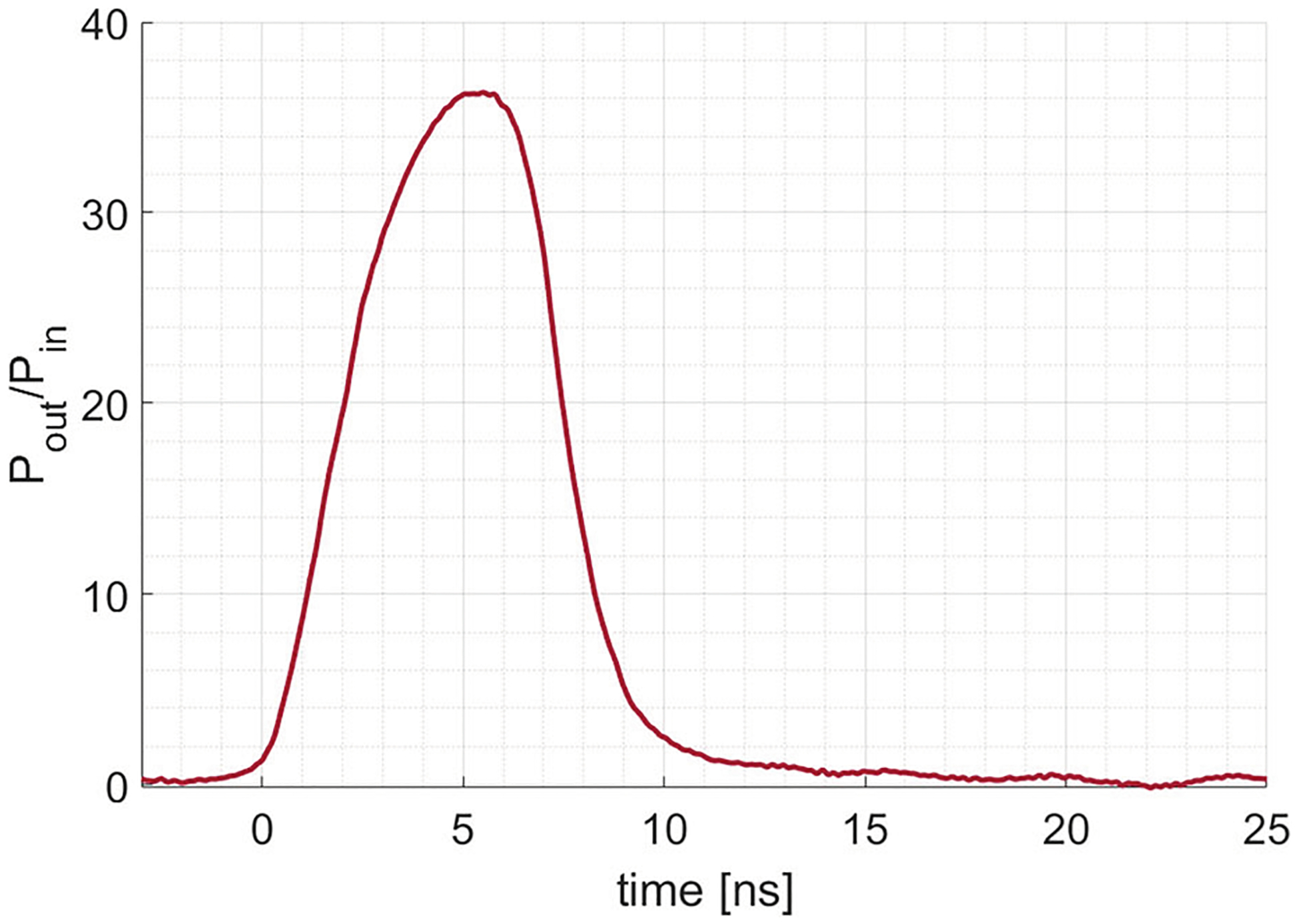
Experimentally measured gain. The value for $P_{\text{out}}$ is obtained from Diode 2 when the ring resonator is filled and triggered with the laser, while the value for $P_{in}$ is obtained from replacing both the Silicon switch and the input coupler with 100% reflective aluminum mirrors

Diode 2 measures the output from the ring resonator. The output power after triggering the LDSS is compared with the source power to obtain the ring resonator gain. The results are shown in Fig. 4. The peak gain reaches a value of 36 ± 1. This value is obtained with the laser operating at its maximum energy of 230 mJ, corresponding to an energy density of 1.3 mJ/cm^2^.

The reflectance does not rise instantaneously and does not reach 100% within the lifetime of the output pulse. The measured gain is therefore lower than the ideal gain achieved inside the ring resonator. To obtain more information on the ring characteristics, we need to know the time dependence of the reflectance during the output of the ring resonator. Therefore, we have both calculated the time dependence of the reflectance and measured it experimentally.

The theoretical reflectance during the output phase of the ring resonator is calculated with an algorithm using the LDSS properties. A LDSS works by changing the reflectance (R), transmittance (T), and absorptance (A) of a semiconductor wafer when illuminating it with laser light of large enough energy density. During and shortly after laser illumination, the wafer transmittance decreases, and its reflectance increases. The specific time-dependent characteristics depend on the laser energy density as well as the laser temporal pulse shape. The details of the theory of the R, T, A of a LDSS as well as experimental data from a 250 GHz LDSS are presented in [26].

**Fig. 5:**
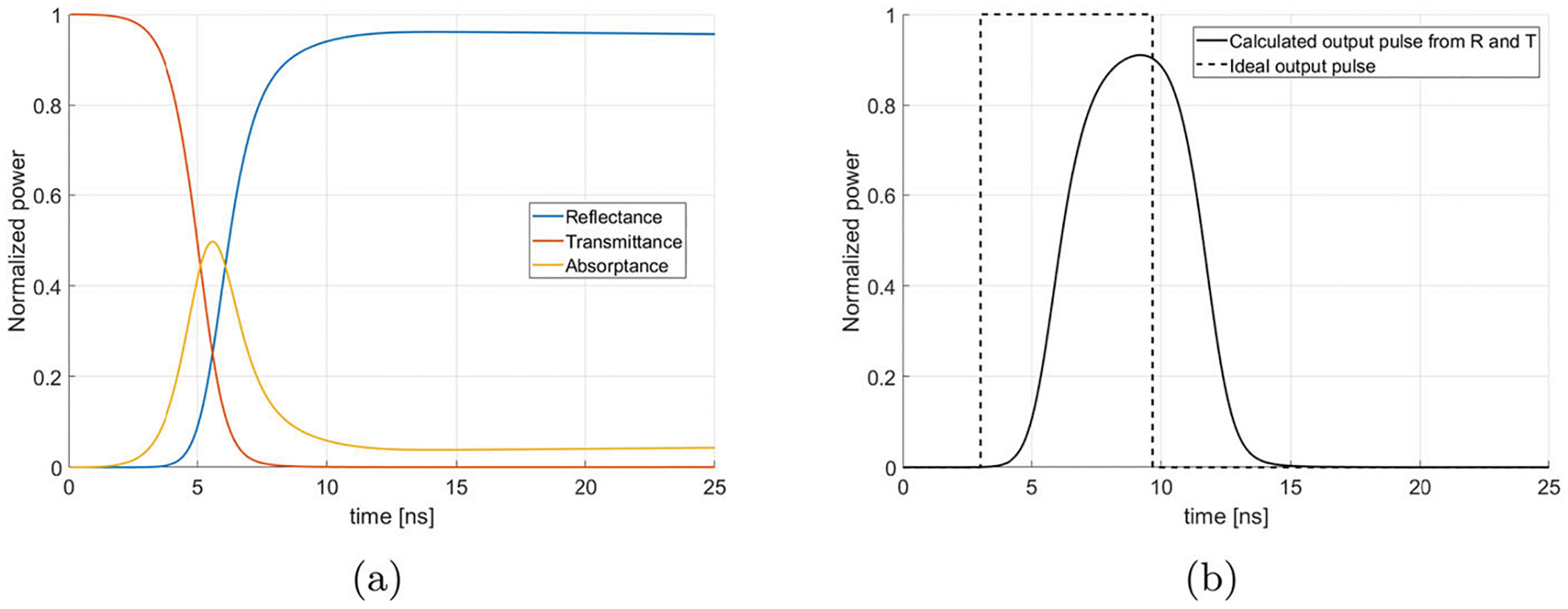
**a** Theoretical reflectance, transmittance, and absorptance of the silicon wafer as a function of time. **b** Ideal (dashed line) and calculated (solid line) output pulse obtained from the theoretical reflectance and transmittance of the semiconductor switch for a 2 m long ring resonator with a laser energy density of 0.5 mJ/cm^2^

This section uses the theoretical reflectance, transmittance, and absorptance of a LDSS to calculate the output pulse time evolution from the quasi-optical ring resonator. The time-dependent values of R, T, and A have been calculated for a 110 GHz LDSS using the theory of [26]. The results are shown in Fig. 5a.

An algorithm is employed to reproduce the physical behavior of the RF wave inside the ring resonator after it has reached saturation. At saturation, the input power from the steady state source is equal to the power loss in the ring as well as the power transmitted out of the input coupler, as described by Eq. 1 for large values of t. The power in the ring is therefore a constant value. When the semiconductor switch is illuminated, a portion of the steady state signal is reflected out of the ring proportional to the increasing switch reflectance, R(t), as shown in Eq. 7.

(7) $$P_{\text{out}}(t)=R(t)P_{\text{ring}}\;\text{for}\;0<t<\tau_{\text{loop}}$$

where $P_{\text{out}}(t)$ is the power output during the first loop after the laser triggers. A loop is defined as the time it takes for the RF wave to cycle back to its original position in the ring. This round trip time is defined as

(8) $$\tau_{\text{loop}}=\frac{L}{c}$$

with a value of $\tau_{\text{loop}}=6.7\;\text{ns}$ for our ring resonator. In the case of this algorithm, $P_{\text{ring}}=1$, and a normalized output shape is calculated. During the first loop, the RF signal that is not reflected out is transmitted through, T(t), or absorbed in the switch as A(t)=1-R(t)-T(t). The transmitted portion will go through a second loop before interacting with the semiconductor switch again.

During the second loop, the power in the ring is no longer constant and is instead determined by what was transmitted through the output coupler in the first loop, neglecting the now small contribution from the RF source through the highly transmissive input coupler as well as the round trip loss. The output of the second loop will therefore be the leftover from the first loop $T\left(t-\tau_{\text{loop}}\right)$ multiplied by the present reflectance of the switch, such that

(9) $$P_{\text{out}}(t)=T\left(t-\tau_{\text{loop}}\right)R(t)\;\text{for}\;\tau_{\text{loop}}<t<2\tau_{\text{loop}}$$

This process is then repeated n times, such that

(10) $$P_{\text{out}}(t)=T\left(t-n\tau_{\text{loop}}\right)T\left(t-(n-1)\tau_{\text{loop}}\right)\cdots T\left(t-\tau_{\text{loop}}\right)R(t)\;\text{for}\;(n-1)\tau_{\text{loop}}<t<n\tau_{\text{loop}}$$

Eventually, the power remaining inside the ring becomes negligible.

In the ideal ring resonator, the switch becomes instantly 100% reflective and no power is lost through absorptance and no power is leaked back into the ring through a finite transmittance. In that scenario, the output of the ring is a square wave with the same magnitude as the power inside the ring, lasting for a duration of $\tau_{\text{loop}}$ and the RF wave does not complete more than one loop after the laser is triggered. The square wave for the ideal case is shown in Fig. 5b.

In a realistic case, the finite laser rise time characteristic of a Q-switched laser causes the reflectance to increase over a few nanoseconds and to reach a value less than 100%. This creates a longer output pulse of a lesser average magnitude than the ideal case, as shown in Fig 5b.

**Fig. 6:**
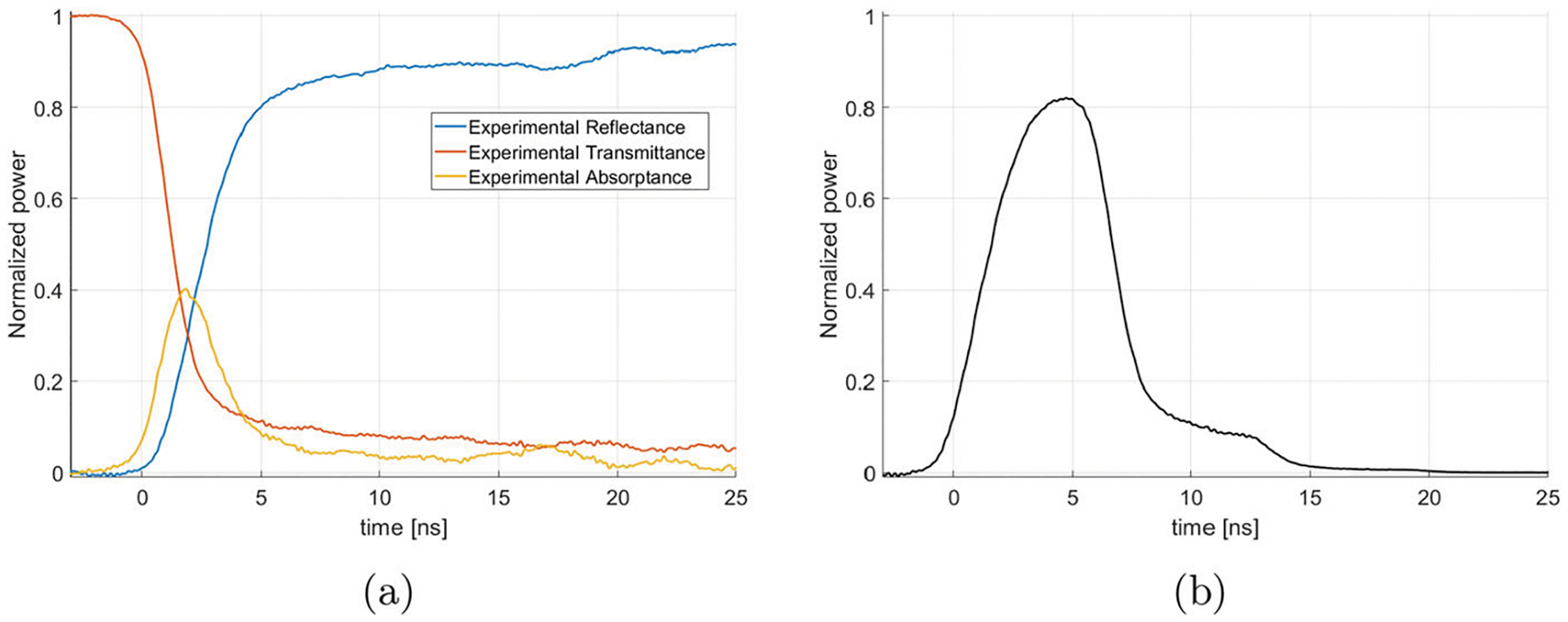
**a** Experimental reflectance, transmittance, and absorptance of the silicon wafer used with the ring resonator at 110 GHz and **b** power output as a fraction of ring internal power calculated from the algorithm described in Sect. 2.2 with a laser energy density of 1.3 mJ/cm^2^. A maximum normalized power of 0.82 ± 0.03 is obtained at 5 ns, meaning that the effective reflectance of the switch employed in the 2 m ring resonator is 0.82 ± 0.03

The theoretical process described above is applied to our experimental data. First, we show our experimentally measured values at 110 GHz of the reflectance, transmittance, and absorptance of the switch in Fig. 6a measured using techniques described in [26]. This information is then used to calculate the output pulse shape using the theory described above, as shown in Fig. 6b.

Figure 6 shows that even though the reflectance of the switch eventually reaches 90%, the peak power output only reaches 82% of its maximum. This is due to the power having been extracted before the switch could reach its peak reflectance, as a result of the finite risetime of the laser pulse.

Now that we know that the switch only reaches a peak reflectance of 82%, we can use the internal gain of 38 ± 4 calculated from Diode 1 in Sect. 2.1 to calculate what would be the output based on the ring characteristics measured from Diode 1. The peak gain is calculated as

(11) $$P_{\text{out},\text{peak}}=R_{\text{peak}}P_{\text{ring}}$$

where $P_{\text{ring}}=38\pm 7$ (obtained from Eq. 6) and $R_{\text{peak}}=0.82\pm 0.03$. This peak gain has a value of $P_{\text{out},\text{peak}}=31\pm 6$. This calculated value is a bit smaller than the measured peak gain of 36 ± 1 obtained from Diode 2 as shown in Fig. 4, but the error bar on the value of $P_{\text{out},\text{peak}}$ is large.

### 2.3 Laser Energy Variation Effect on Output Pulse

The previous section showed a maximum gain of 36 achieved at the maximum laser energy. However, there are many advantages to running ring resonators with lower laser energy, such as increased safety, decreased cost, and increased rep-rate capabilities. The lower laser energy comes at a cost on both the peak effective reflectance, which leads to lower total gain, and a change in the output pulse shape.

**Fig. 7:**
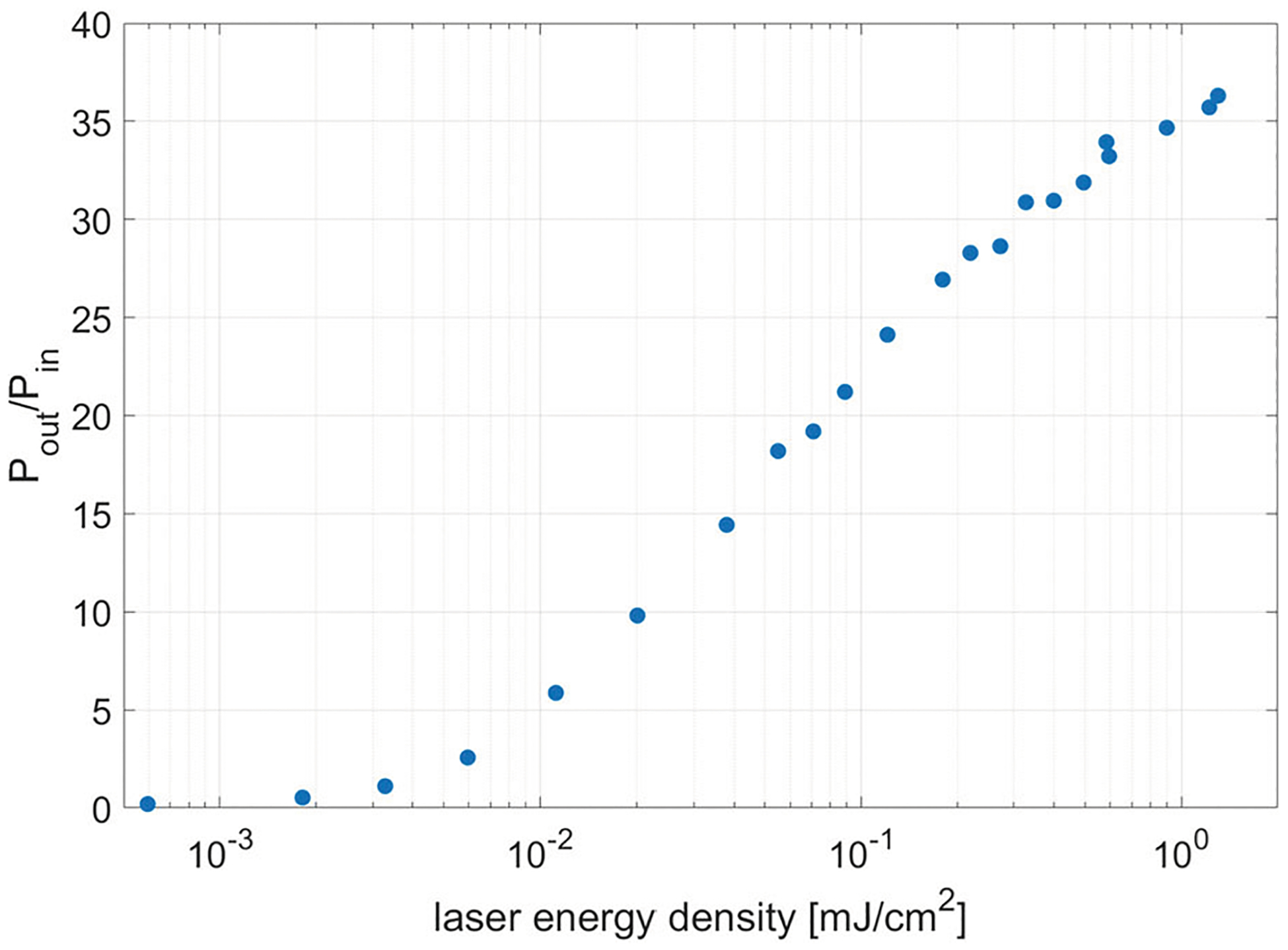
Peak measured gain as a function of laser energy density

The effect on peak gain is shown in Fig. 7. The total gain falls off with laser energy density, as there are now insufficient electron–hole pairs to reach the critical plasma density to effectively reflect the RF wave. This decrease in gain is explained by the lowering of the reflectance of the wafer for lower laser energy density.

The present experiment does not reach the maximum possible gain, as the saturation of the curve of Fig. 7 is not reached. This is due to the large size of the 150 mm diameter wafer, designed this way to avoid air breakdown when used with high power. A more powerful laser would have been necessary to reach the maximum possible reflectance, hence maximum gain.

**Fig. 8:**
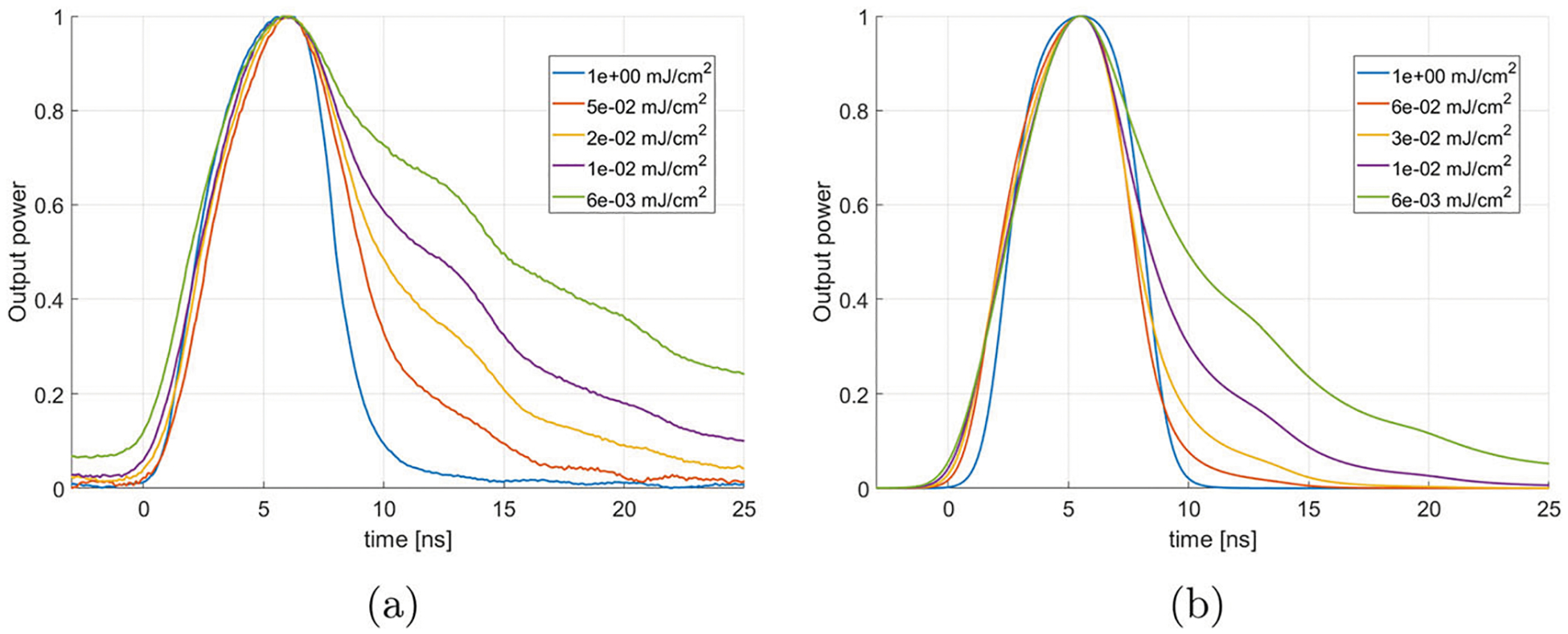
Ring resonator output normalized to its peak value. **a** Experimental measurements and **b** theoretical calculation obtained from the algorithm in Sect. 2.2 derived from the LDSS theory. The features of the long trailing edge are reproduced by the theory, although at a lower laser energy density

Another consequence of the lowering of the laser energy is the change in the output pulse time trace. Specifically, the pulse becomes broader. This is due to the increase in transmittance after the laser has fired, allowing a significant portion of the power in the ring to stay trapped for many additional loops, therefore stretching the output over a longer period of time. This is illustrated in Fig. 8 where the normalized output pulse shapes are shown at different laser energy, both for the direct experimental measurement, and for the calculated pulse derived from the theory shown in Sect. 2.2.

**Fig. 9:**
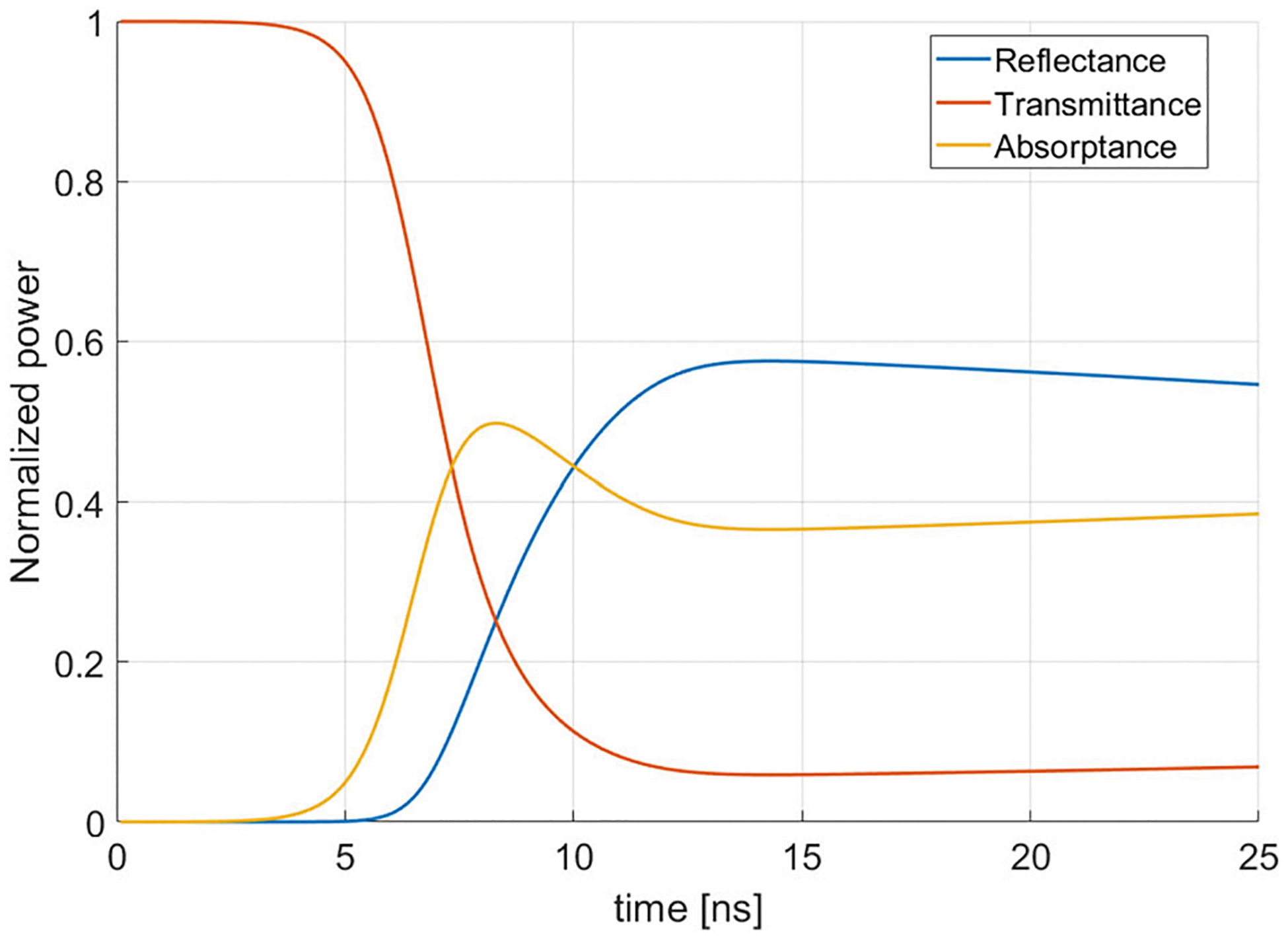
Theoretical reflectance, transmittance, and absorptance at low laser energy, 0.03 mJ/cm^2^. The transmittance remains at a finite value, although most of the energy is lost through absorptance in the wafer

The main feature is the appearance of the trailing edge of the pulse when the laser energy density falls below 1 mJ/cm^2^. This is present in both the theory and the experiment. This is due to the transmittance staying finite after the end of the laser pulse, as shown in Fig. 9. In that case, the transmittance reaches a value close to zero at high energy density, but stays finite at lower energy density, permitting a significant portion of the internal ring power to stay trapped. This trapped portion is then extracted over subsequent loops, therefore increasing the pulse length while decreasing the peak pulse magnitude.

### 2.4 Energy Efficiency

For high repetition rate application, the total energy efficiency of the ring resonator is important. The energy efficiency is defined as the ratio of the energy contained in the ring over the total energy output from the source, that is

(12) $$\eta =\frac{P_{\text{ring}}\tau_{\text{loop}}}{P_{\text{inc}}t}$$

which, using Eq. 1, simplifies to

(13) $$\eta =\frac{4Q_{tot}^{2}}{\omega_{0}Q_{c}t}{\left(1-\text{exp}\left(-\frac{\omega_{0}t}{2Q_{tot}}\right)\right)}^{2}$$

**Fig. 10:**
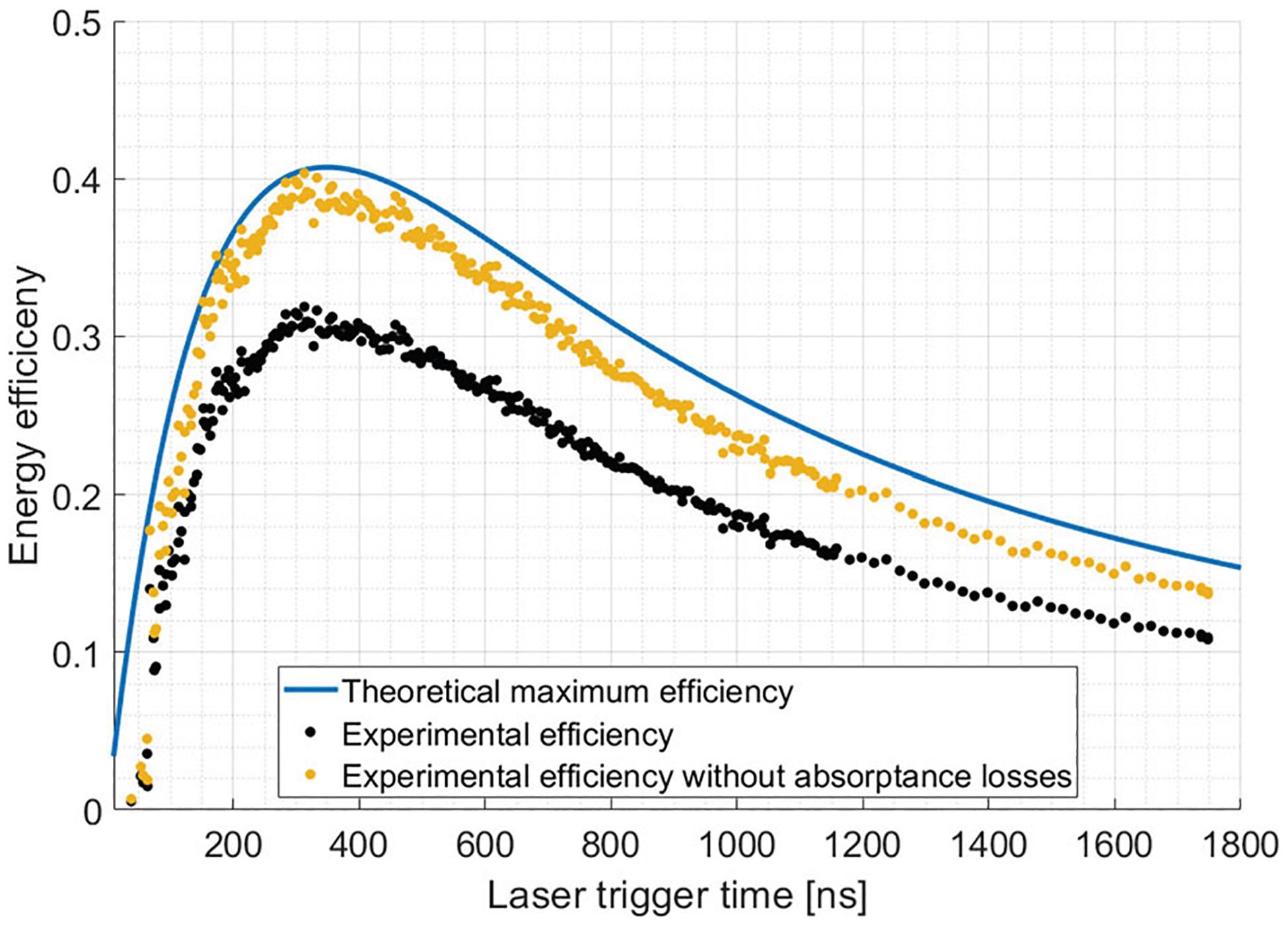
Energy efficiency as a function of the laser trigger time with respect to the beginning of the charging of the ring resonator. The solid line is the efficiency as calculated from Eq. 13 while the black dots are experimental data points measured using Diode 2 for a laser fluence of 1.3 mJ/cm^2^. The yellow points are the experimental data scaled using a factor of 0.79 which removes the contribution of the wafer absorptance to the energy loss. Note that the x axis starts at t = 10 ns in order to avoid the high values of experimental efficiency calculated when the denominator in Eq. 12 approaches zero. The theoretical efficiency converges to zero at t = 0

Equation 13 is maximized at $t=1.256\frac{2Q_{\text{tot}}}{\omega_{0}}$. Note that this maximum is obtained before the ring is fully saturated, meaning that the optimal operating point sacrifices the value of gain that can be obtained. The result of this equation as well as the comparison with experimental measurement is shown on Fig. 10. The maximum obtainable efficiency is calculated using Eq. 13 evaluated at the maximum. This value is

(14) $$\eta_{max}=0.814\frac{Q_{\text{loss}}/Q_{c}}{Q_{\text{loss}}/Q_{c}+1}$$

At critical coupling, $Q_{\text{loss}}=Q_{c}$, the maximum efficiency is $\eta_{max}=0.407$. The maximum possible efficiency of η=0.814 is obtained when $Q_{\text{loss}}/Q_{c}\to \infty$. However, according to Eq. 6, this condition also guarantees that the gain converges to zero. Therefore, it is only possible to increase the efficiency of the ring resonator by reducing the ring losses (increasing $Q_{\text{loss}}$) and increasing the input coupler transmittance (reducing $Q_{c}$), but that increase comes at the cost of reducing the maximum gain.

The blue curve of Fig. 10 shows the calculated efficiency of our ring resonator as a function of the laser trigger time assuming a 100 % reflecting, lossless output coupler. The curve is maximized at around 350 ns, in agreement with Eq. 13 for our 2 m ring resonator using the calculated Q values of Sect. 2.1. The maximum efficiency is reached before the ring is fully saturated. The theoretical curve is compared with experimental data, shown with black dots. We observe that the peak efficiency is much lower for our experiment compared with the theoretical maximum. The discrepancy is explained by the absorption losses in the LDSS.

The energy loss from absorption is calculated using the experimental reflectance, transmittance, and absorptance data of Fig. 6b. The ratio between the total energy from the curve calculated in Fig. 6b and the total energy from the ideal output pulse of Fig. 5b is given by the following equation

(15) $$\frac{E_{\text{output}}}{E_{\text{ideal}}}=\frac{\int_{0}^{\infty }\;\;P_{\text{out}}(t)dt}{P_{\text{ring}}\tau_{\text{loop}}}$$

where $P_{\text{out}}(t)$ is the time-dependent output pulse calculated in Fig. 6b and $P_{\text{ring}}=1$ in the context of that algorithm. We calculate this ratio to be 0.79. The experimentally measured efficiency divided by this factor is shown by the yellow dots in Fig. 10. This curve now closely follows the theoretical efficiency. Therefore, most of the difference in theoretical efficiency and measured efficiency is explained by the additional energy loss in the absorptance by the silicon wafer during the output phase of the ring resonator.

## 3 Conclusion

A ring resonator has been successfully designed with critical coupling confirmed by the experimental measurement. The experimental results show that the LDSS theory along with the algorithm developed are a robust way to predict the pulse obtained from a ring resonator of a given length. In particular, this paper shows how measurements of the ring charging characteristics can successfully predict the Q value as well as the values of losses and transparency.

We then analyzed the important effects of changing the laser energy density. The measurements showed that there are compromises on both the sharpness of the output pulse and the maximum gain achievable when using a lower-energy laser pulse. Finally, the energy efficiency of the ring resonator is shown to be limited by the absorptance of the LDSS.

The results from this design and analysis show that it is possible to tailor an output high-power pulse by careful selection of the laser energy density, the semiconductor characteristics, and the ring resonator total length. A fast rise time (subnanosecond) and high energy density laser will result in a sharp output pulse, while a slower and lower energy laser creates stretched pulses. However, much of the output power will be lost through the absorptance term in the LDSS when using too low of a laser energy. This will seriously limit the attainable efficiency of the system.

Measuring the precise losses and transparency of a ring resonator can be challenging. The techniques shown in Sect. 2.1 can be employed to verify the design values without having to align and handle a high-power laser and without a high-power millimeter-wave source. It is therefore possible to easily tune a ring resonator to attain critical coupling by changing the input coupler to one that has the same transmittance as the measured internal losses. Moreover, the design is further helped by the algorithm which can calculate an output pulse shape knowing only the ring resonator length as well as the LDSS characteristics vs. time.

This present experiment could be easily adapted to automate a temperature compensation system for the ring length. This is possible thanks to the alignment stage which is already controlled electronically. Moreover, the large beam size of the resonator permits the use of a MW-level input beam without air breakdown. Those two characteristics can lead to a stable, high-power ring resonator able to operate without a special temperature-controlled base as well as without a dedicated vacuum chamber. This opens the path to novel, high-power, and high-frequency linear accelerator concepts.

## Data Availability

The datasets generated during the current study are available from the corresponding author upon reasonable request.
